# Rotator cuff sparing approach for shoulder arthroplasty

**DOI:** 10.1016/j.xrrt.2026.100738

**Published:** 2026-03-28

**Authors:** Arnaud Walch, Adrien Jacquot, Sophie Grosclaude, Jean-David Werthel

**Affiliations:** a Hôpital Edouard Herriot, Lyon, France; b Centre ARTICS, Chirurgie des Articulations et du Sport, Essey-les-Nancy, France; c Clinique du Parc, Lyon, France; d Hopital Ambroise Paré, Boulogne-Billancourt, France

**Keywords:** Rotator cuff sparing, Shoulder arthroplasty, Subscapularis sparing, Rotator interval, Surgical technique, Minimally invasive surgery

Subscapularis (SSC) management is a critical aspect of shoulder arthroplasty. Several techniques are available, including SSC peel, SSC tenotomy, and lesser tuberosity osteotomy (LTO). Del Core et al^4^ reported healing rates of 74.1% for the SSC peel and 75.9% for SSC tenotomy, compared with a significantly higher healing rate of 95.8% following LTO.

The concept of a SSC-sparing approach for anatomic total shoulder arthroplasty (aTSA) was first reported by Lafosse et al,^9^ who used a superolateral approach and accessed the glenohumeral joint entirely through the rotator interval without dislocating the humeral head. This technique preserved the SSC but required elevation of the anterior deltoid and an acromioplasty and did not allow systematic removal of inferior humeral osteophytes. Morishige et al^10^ subsequently described a SSC-sparing deltopectoral (DP) approach in which an L-shaped capsulotendinous flap of the inferior one-third of the SSC is elevated while the superior fibers remain attached to the lesser tuberosity. Ding et al^5^ later proposed a DP complete SSC-sparing technique for aTSA using a small window beneath the SSC to resect inferior humeral osteophytes but reported a substantial rate of residual osteophytes because visualization was insufficient.

For reverse total shoulder arthroplasty (rTSA), Lädermann et al and Chung et al described SSC- and deltoid-preserving DP approaches that allow early active motion without post-operative immobilization.^1^^,^^8^ More recently, Jacquot et al^7^ refined this concept with the anterior muscle-sparing (AMS) DP approach that may be use with standard TSA instrumentation, adding a larger inferior window that enables complete removal of inferior humeral osteophytes and an extensive inferior capsular release on the humeral side while preserving the pectoralis major and SSC. However, in the setting of an intact rotator cuff, Jacquot et al recommend performing a supraspinatus tenotomy in order to preserve the SSC. Mighell et al further developed the SSC-sparing windowed anterior technique for aTSA with an intact cuff, combining an inferior window and a rotator interval working portal, but this technique relies on dedicated instrumentation and an intramedullary cutting guide.^12^^,^^13^

We describe technical refinements of the AMS approach that allow exposure of both the proximal humerus and the glenoid through the rotator interval in shoulders with an intact rotator cuff undergoing shoulder arthroplasty. These modifications allow implantation of the prosthesis entirely through a DP approach through the rotator interval and can be performed using standard TSA instrumentation.

## Technique

The approach is similar to a standard DP exposure, with specific modifications that provide sufficient mobilization of the SSC and supraspinatus, and visualization of the proximal humerus:

- Patient positioning: the patient is placed in a beach-chair position, with the operative shoulder positioned more laterally at the edge of the table to allow the degree of adduction required to dislocate the humeral head through the rotator interval.
- Intraoperative muscle relaxation is mandatory to facilitate humeral mobilization and capsular release.
- Extended rotator interval resection and anterior capsulectomy: the rotator interval, including the coracohumeral ligament, is widely excised, and an extensive anterior capsulectomy is performed through the interval.
- Inferior capsulotomy through an inferior window between the SSC and the latissimus dorsi: an inferior capsulotomy is carried out with section of the inferior glenohumeral ligament and the inferior capsule on both the glenoid and humeral sides.

These last 2 steps provide sufficient release of the SSC, without detaching it, to allow external rotation and to translate the SSC superiorly for inferior humeral exposure to remove osteophytes and inferiorly for glenoid exposure.

- Humeral exposure: a retractor is passed through the supraspinatus muscle belly from its deep surface to its superficial aspect. This maneuver allows retraction of the supraspinatus, thereby facilitating exposure of the posterior aspect of the humeral head.

The patient is placed in the beach-chair position, with the shoulder at the edge of the table to allow adduction and retropulsion, and the arm placed in an arm holder.

A standard DP skin incision is used. The DP interval is developed, the cephalic vein is retracted laterally, and the conjoint tendon is mobilized and retracted medially. The superior 1 cm of the pectoralis major tendon is incised to increase exposure.

With the arm in internal rotation and the elbow at the side, the lateral border of the SSC is identified using the long head of the biceps tendon as a landmark.

The rotator interval is then identified. To do so, the arm is placed in external rotation and retropulsion, the coracoacromial ligament is resected, and the rolled superior border of the SSC and the anterior border of the supraspinatus are visualized and released from the capsule, and all tissues contained within the rotator interval between these 2 landmarks are excised.

**Figure 1:**
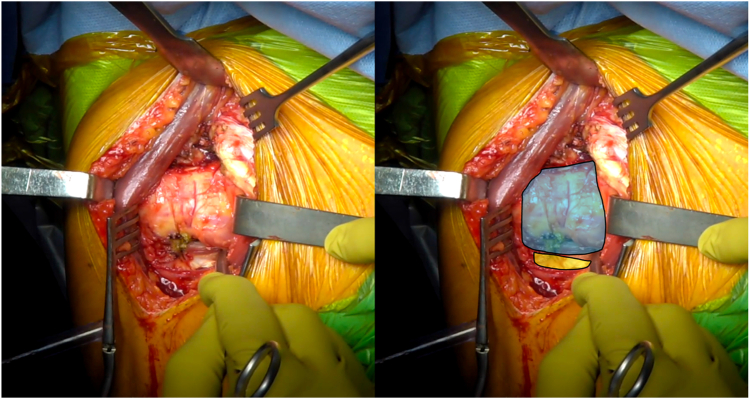
Identification of the inferior subscapularis border. The tendon of the latissimus dorsi is bluntly dissected and allows identification of the inferior border of the SSC muscle. *Blue* indicates the subscapularis muscle, and *yellow* indicates the latissimus dorsi tendon. *SSC*, subscapularis.

**Figure 2:**
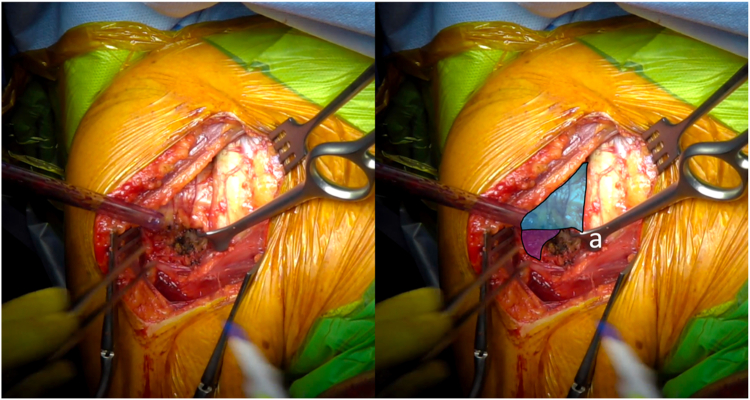
(*a*) A small Hohmann retractor is inserted to elevate the subscapularis muscle. *Blue* indicates the subscapularis muscle, and *purple* indicates the humerus.

**Figure 3:**
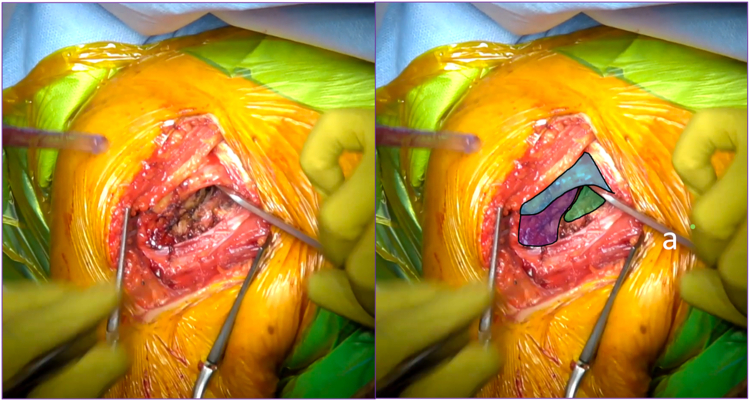
(*a*) A double-bent Hohmann retractor is passed under the subscapularis muscle and supported on the anterior rim of the glenoid. *Blue* indicates the elevated subscapularis muscle, *purple* indicates the humerus, and *green* indicates the glenoid.

Then the arm is placed in external rotation with the elbow at the side. The anterior humeral circumflex artery and its 2 accompanying veins (“three sisters”) are coagulated. The tendon of the latissimus dorsi is bluntly dissected and allows identification of the inferior border of the SSC muscle (Fig. 1). Mayo scissors are then advanced between the capsule and the deep surface of the SSC, and a small Hohmann retractor is inserted to elevate the inferior part of the SSC (Fig. 2). The inferior capsule is resected along the calcar, staying in contact with the humeral neck to avoid any injury to the axillary nerve. A double-bent Hohmann retractor is then passed under the SSC and supported on the anterior rim of the glenoid (Fig. 3). This allows an inferior capsulotomy of the glenoid to be performed from 5 o'clock to 7 o'clock, again staying close to bone, and completes the inferior capsular release on the humeral side while positioning the arm in flexion, adduction, and progressive external rotation. Inferior humeral osteophytes are then removed as needed from beneath the SSC.

**Figure 4:**
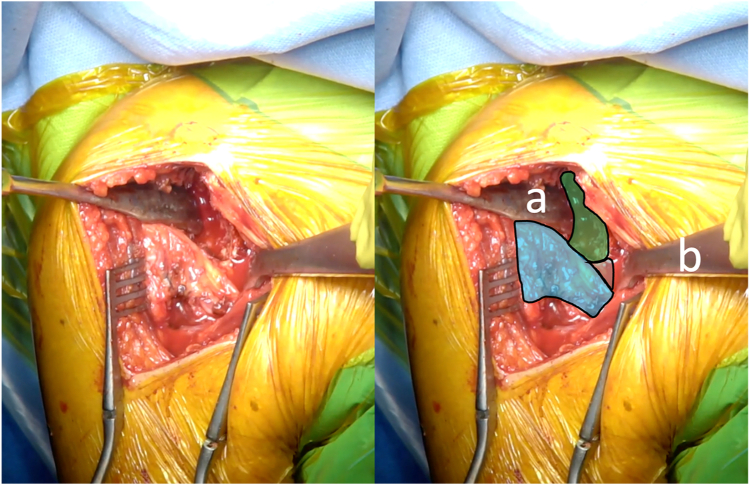
(*a*) A Trillat retractor is inserted into the glenohumeral joint, and (*b*) a double-bent Hohmann is placed above the superior border of the SSC along the anterior rim of the glenoid. *Blue* indicates the lowered subscapularis muscle, *green* indicates the glenoid, and *gray* indicates the anterior capsule and middle glenohumeral ligament. *SSC*, subscapularis.

The arm is then positioned in internal rotation with the elbow at the side. A Trillat or a Fukuda retractor can then be inserted into the glenohumeral joint, and a double-bent Hohmann is placed above the superior border of the SSC, along the anterior rim of the glenoid (Fig. 4). This configuration allows complete skeletonization of the coracoid base, with excision of all residual coracohumeral ligament and a complete anterior capsulectomy from approximately 11 o'clock to 5 o'clock and a release of the deep surface of the SSC.

**Figure 5:**
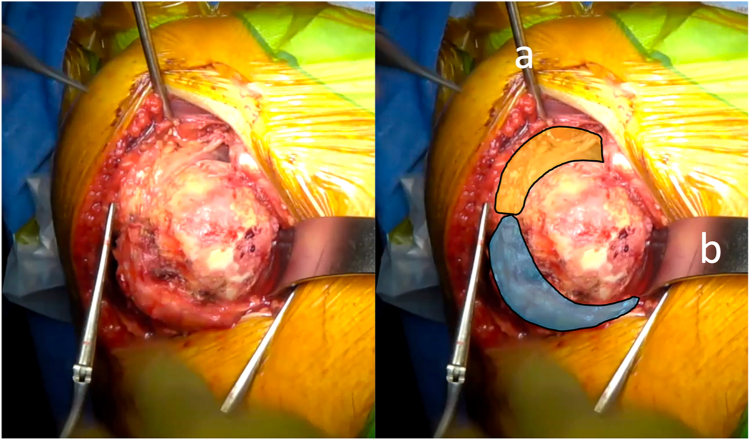
The humeral head is delivered through the rotator interval by placing the arm in adduction, retropulsion, and external rotation. (*a*) A pointed Steinmann pin is passed through the muscle belly of the supraspinatus from its deep surface to the outside, providing a handle to retract the supraspinatus posteriorly. This maneuver allows for visualization of the posterior cuff insertion. (*b*) A large curved retractor is positioned under the calcar to depress the SSC and protect the axillary nerve. *Blue* indicates the subscapularis muscle, and *orange* indicates the posterior cuff. *SSC*, subscapularis.

Once the anterior capsular release is complete, the humeral head can be delivered through the rotator interval by placing the arm in adduction, retropulsion, and external rotation. The long head of the biceps is resected. A pointed Steinmann pin is passed through the muscle belly of the supraspinatus from its deep surface to the outside, providing a handle to retract the supraspinatus posteriorly while preserving the supraspinatus tendon (Fig. 5). This maneuver allows for visualization of the posterior cuff insertion. A large curved retractor is positioned under the calcar to depress the SSC and protect the axillary nerve. Humeral head osteotomy and preparation of the humeral canal are then performed according to the chosen implant system.

**Figure 6:**
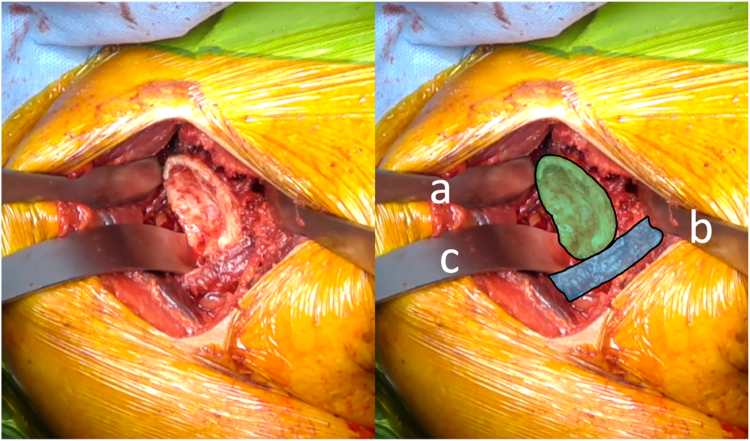
The arm is placed in antepulsion and internal rotation. (*a*) A double-bent Hohmann retractor is placed posterosuperiorly on the glenoid neck. (*b*) A second double-bent Hohmann retractor is positioned on the anteroinferior glenoid to depress the SSC, and (*c*) a large curved retractor is positioned posteroinferiorly to push the humerus down and posterior to the glenoid. This three-retractor construct provides sufficient “en face” visualization of the glenoid. *Blue* indicates the subscapularis muscle, and *green* indicates the glenoid. *SSC*, subscapularis.

Glenoid preparation is carried out next. The arm is placed in antepulsion and internal rotation. A double-bent Hohmann retractor is placed posterosuperiorly on the glenoid neck. A second double-bent Hohmann retractor is positioned on the anteroinferior glenoid to depress the SSC, and the large curved retractor is positioned posteroinferiorly to push the humerus down and posterior to the glenoid. This three-retractor construct (similar to what is routinely used for glenoid exposure after SSC takedown) provides sufficient en face visualization of the glenoid to complete the posterior glenoid capsulotomy and to perform standard preparation and implantation of the baseplate or glenoid component (Fig. 6).

For rTSA, all retractors are removed after glenoid preparation and baseplate implantation, and a single inferior retractor is left at the lower glenoid rim to facilitate glenosphere implantation.

The humerus is again delivered through the rotator interval, as during the initial preparation, to allow implantation of the definitive humeral component. Fluoroscopic controls of humeral component positioning and of complete removal of the osteophytes can be performed. The prosthesis is then reduced. In the case of a rTSA, a bone hook can be used as a lever to gently bring the humerus down and achieve reduction. The rotator interval is closed, and the superior portion of the pectoralis major tendon is then repaired while performing a biceps tenodesis onto its upper border. The wound is closed in layers without a drain.

Post-operatively, patients are instructed to wear a sling for comfort. Post-operative rehabilitation is similar to that used after a standard approach. Early active use of the arm is encouraged as tolerated, without formal restrictions beyond pain.

## Discussion

We describe technical refinements of the previously published AMS approach technique that allow a sufficient humeral and glenoid exposure to perform a shoulder arthroplasty in the setting of an intact rotator cuff while preserving all rotator cuff tendons, and using standard TSA instrumentation. The refinement introduced beyond the original AMS technique primarily concerns humeral exposure when the rotator cuff is intact. This is achieved by passing a pointed Steinmann pin through the supraspinatus muscle belly from its deep surface to its superficial aspect. This maneuver allows controlled posterior retraction of the supraspinatus, thereby facilitating exposure of the posterior aspect of the humeral head.

This approach is primarily intended for primary shoulder arthroplasty in patients with an intact rotator cuff. The usefulness of SSC-sparing in rTSA is debated,^2^, ^3^, ^4^^,^^6^ but it appears particularly well suited for aTSA and hemianatomic arthroplasty.^5^^,^^9^ However, regardless of the implant used, the advantage of this technique is that it eliminates the risk of secondary tendon rupture while providing adequate humeral and glenoid exposure to place the implants. This is particularly valuable in elderly patients, who may live alone and cannot manage daily activities with an immobilized arm, as well as in younger patients, in whom preservation of an intact rotator cuff is a priority.

Relative contraindications include severe pre-operative stiffness, major glenoid deformity, revision surgery, and post-traumatic cases because of the stiffness. In such situations, conversion to a standard DP approach with SSC takedown may be necessary to ensure adequate visualization and implant positioning. Intraoperatively, in the presence of a stiff shoulder or if adequate exposure cannot be safely achieved, the surgeon should not hesitate to detach the SSC—whether by peel, tenotomy, or LTO—rather than risking an intramuscular or musculotendinous rupture due to excessive traction.

At the 6 o'clock position of the glenoid, Price et al^11^ reported a mean distance of 12.4 mm between the axillary nerve and the glenoid rim. This anatomical consideration should be kept in mind when performing the inferior capsulotomy, and the surgeon should remain strictly bone-adjacent, as in a standard DP approach.

Another concern relates to elderly patients who may have a weakened tendon–bone enthesis, in whom intraoperative manipulation should be particularly cautious. A high-risk step in rTSA occurs after implantation of the baseplate and glenosphere, during humeral implantation. At this stage, the glenohumeral offset is maximal, and traction places the rotator cuff at risk. Once the prosthesis is reduced, the glenohumeral offset decreases, resulting in lower muscular tension.

This approach does not require any specific instrumentation: humeral preparation can be performed with standard instruments that come from above the humerus, as in a lateral approach, or from anterior to the humeral head. Likewise, no dedicated retractors are mandatory. However, we recommend the use of a large curved retractor, which provides satisfactory exposure. A mechanical arm holder is also, in our view, important. As in any shoulder surgery, humeral position is critical for exposure: the arm holder maintains the humerus in the desired position when delivering the head through the rotator interval and facilitates glenoid exposure by placing the arm in forward flexion and internal rotation.

As with any surgical technique, there is a learning curve, and certain adaptations can be introduced to facilitate its mastery. Section of the coracoacromial ligament as well as the superior aspect of the pectoralis major tendon may improve exposure at the beginning of the learning curve. However, these structures can be preserved. Because the SSC limits external rotation and is, by definition, present in the SSC-sparing approaches, a common limitation of previously described techniques is the difficulty in obtaining sufficient external rotation, which is essential to expose the humerus. The amount of external rotation required depends primarily on the humeral preparation instrumentation. If the humeral guides are designed to be used from above the humerus, only limited external rotation is necessary. Conversely, if the instrumentation needs to be introduced from anterior to the humeral head, a large degree of external rotation is required. Consequently, all of the published SSC-sparing techniques rely on humeral preparation performed from above the humerus because it is more adapted. We recommend, during the learning curve of SSC-sparing techniques, using systems that allow humeral preparation from above.

**Figure 7:**
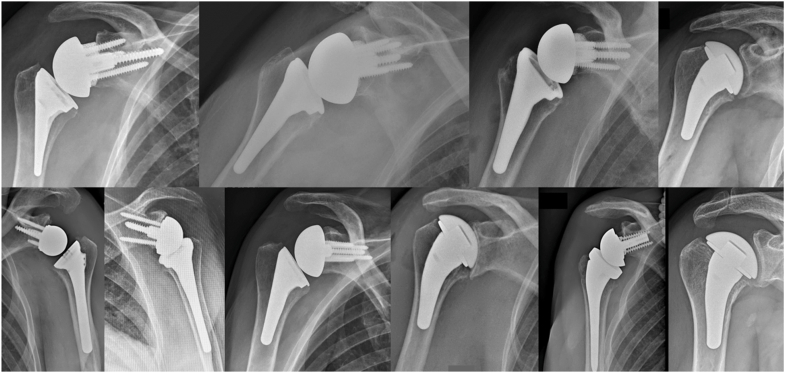
Post-operative radiographs of the first 10 cases treated with this approach.

The main limitation of this work is the lack of reported clinical outcome data. The technique was applied in 10 patients without intraoperative conversion to a SSC takedown or implant malposition (Fig. 7). However, this limited experience does not allow any inference regarding clinical benefit, complication rates, or generalizability. Further prospective studies with formal clinical evaluation are necessary before any conclusions can be drawn.

## Conclusion

These technical refinements allow exposure of both the proximal humerus and the glenoid through the rotator interval in shoulders with an intact rotator cuff undergoing shoulder arthroplasty. Proximal humeral exposure is adequate to implant the prosthesis, and inferior humeral osteophytes can be resected when present. Glenoid exposure is likewise satisfactory and allows glenoid lateralization if desired by the surgeon.

## Disclaimers

Funding: No funding was disclosed by the authors.

Conflicts of interest: Dr. Adrien JACQUOT is a paid consultant for ARTHREX, not related to the subject of this article.

Dr. Arnaud Walch is a paid consultant for Stryker, related to the subject of this article.

Dr. Sophie GROSCLAUDE declare to be a paid consultant for ENOVIS and LEPINE, related to the subject of this article.

Dr. Jean-David Werthel is a paid consultant for Stryker and receives royalties from Stryker.
